# Physicians' use of the 5As in counseling obese patients: is the quality of counseling associated with patients' motivation and intention to lose weight?

**DOI:** 10.1186/1472-6963-10-159

**Published:** 2010-06-09

**Authors:** Melanie Jay, Colleen Gillespie, Sheira Schlair, Scott Sherman, Adina Kalet

**Affiliations:** 1 New York University School of Medicine, Division of General Internal Medicine, New York, NY, USA; 2 Montefiore Medical Center, Division of General Internal Medicine, Bronx, NY, USA; 3 New York Harbor VA, New York, NY, USA

## Abstract

**Background:**

Physicians are encouraged to counsel obese patients to lose weight, but studies measuring the quality of physicians' counseling are rare. We sought to describe the quality of physicians' obesity counseling and to determine associations between the quality of counseling and obese patients' motivation and intentions to lose weight, key predictors of behavior change.

**Methods:**

We conducted post-visit surveys with obese patients to assess physician's use of 5As counseling techniques and the overall patient-centeredness of the physician.. Patients also reported on their motivation to lose weight and their intentions to eat healthier and exercise. One-way ANOVAs were used to describe mean differences in number of counseling practices across levels of self-rated intention and motivation. Logistic regression analyses were conducted to assess associations between number of 5As counseling practices used and patient intention and motivation.

**Results:**

137 patients of 23 physicians were included in the analysis. While 85% of the patients were counseled about obesity, physicians used only a mean of 5.3 (SD = 4.6) of 18 possible 5As counseling practices. Patients with higher levels of motivation and intentions reported receiving more 5As counseling techniques than those with lower levels. Each additional counseling practice was associated with higher odds of being motivated to lose weight (OR 1.31, CI 1.11-1.55), intending to eat better (OR 1.23, CI 1.06-1.44), and intending to exercise regularly (OR 1.14, CI 1.00-1.31). Patient centeredness of the physician was also positively associated with intentions to eat better (OR 2.96, CI 1.03-8.47) and exercise (OR 26.07, CI 3.70-83.93).

**Conclusions:**

Quality of physician counseling (as measured using the 5As counseling framework and patient-centeredness scales) was associated with motivation to lose weight and intentions to change behavior. Future studies should determine whether higher quality obesity counseling leads to improved behavioral and weight outcomes.

## Background

There is consensus that physicians should counsel obese patients to lose weight [1-4]. Physician weight loss counseling is generally positively associated with self-reported behavior change in patients [5-7], but the quality of physician counseling is rarely assessed [8]. Physicians often fail to counsel obese patients [5,9-12] and, when counseling occurs, it is often of poor quality. In one study, of patients who reported discussing their weight, only 5% received diet and exercise advice [6]. Potential reasons why physicians do not adequately counsel patients include lack of time [13], poor training/competency [14-18], and negative attitudes about obesity [19-22].

The 5As counseling framework has been proposed as a way to teach and evaluate the quality of obesity counseling [8,16,23,24]. The 5As framework guides the physician to Assess risk, current behavior, and readiness to change, Advise change of specific behaviors, Agree and collaboratively set goals, Assist in addressing barriers and securing support, and Arrange for follow-up [8,16,23,25]. Training physicians about this framework has been shown to improve patient outcomes in smoking cessation [26]. When combined with office management strategies, training physicians to use the 5As was found to produce greater lipid control and weight loss [27] in patients.

In addition to the 5As model, patient-centeredness is an important measure of the quality of the patient-physician interaction. Patient-centeredness can be defined as being responsive to a patients' needs, beliefs, values, and preferences [28]. An example of a patient-centered counseling strategy would be to discuss weight loss goals in the context of a patients' individual reasons for wanting to lose weight (well-being vs. appearance vs. improvement of diabetes control, etc.). Patient-centeredness is endorsed by patients [29] and associated with increased patient satisfaction and improved health outcomes [30,31].

The purpose of this study was to explore how quality of obesity counseling may lead to behavior change in obese patients. We used adherence to the 5As model and patient-centeredness as our main quality measures. We chose motivation and intention as intermediary patient outcomes since they are key elements of evidence based behavior change models [32] and have been shown to be determinants of behavior change in the setting of weight control [33-35]. In order to determine the potential impact of the quality of physicians' obesity counseling on these variables, we surveyed obese patients immediately after their visit with their physician. We focused on a cohort of residents with varied exposure to training in order to address the questions of whether quality of counseling, regardless of training, is associated with intermediary outcomes. We hypothesized that the quality of physicians' obesity counseling would be associated with higher patient motivation to lose weight as well as stronger intentions to change their diet and exercise behaviors.

## Methods

### Subjects

#### Residents

We included all 23 primary care resident physicians at New York University. They all had consented to be part of a medical education research registry approved by the Institutional Review Board at New York University. The residents were part of a non-randomized study to look at the impact of a 5-hour obesity counseling training on quality of counseling and markers of behavior change in patients. Eleven of the residents received the curriculum and 12 did not. Besides the curriculum, the residency program already emphasized behavior change counseling skills, so there was substantial overlap in the distribution of quality of counseling between the curriculum and non-curriculum groups. Thus, for the purpose of this paper's exploration of links between counseling and patient motivations and intentions, regardless of differences in physician training, the residents and their patients were evaluated in aggregate.

#### Patients and Procedures

We invited all adult, English and Spanish-speaking obese (body mass index(BMI) ≥ 30 kg/m^2^) patients (≥18 years) seen by primary care residents at Gouverneur Healthcare Services to participate in this study. This clinic is part of the New York City Health and Hospitals Corporation (HHC). Gouverneur is located in the Lower East Side of Manhattan and serves a largely low income, underserved immigrant population. Fifteen nursing staff identified English and Spanish-speaking patients with a BMI ≥ 30 kg/m^2^. Research assistants approached these patients immediately after their visit with their physician to invite them to be part of the study. Because low health literacy is common in this setting, all the patients provided verbal consent to be interviewed after listening to a consent script. Research assistants then verbally adminsitered a 30-minute structured survey to each patient in a separate room. They explicitly assured the patients that their physician would not be made aware of the answers. Participants received a pedometer (approximate value = $3.30) as compensation. We excluded patients during analysis if their chief complaint (determined during chart abstraction) indicated they had an acute visit (pain, fever, infectious disease, upper respiratory tract infection, shortness of breath, palpitations, dizziness) as we believe that these immediate needs take priority over obesity counseling, that physicians are therefore not likely to have sufficient time to address obesity in the context of an acute complaint, and that patient's receptivity to obesity counseling is negatively impacted if the patient has discomfort or distress. We also excluded patients during analysis who were incorrectly identified by nursing staff as obese or high risk --i.e. did not have a BMI that rounded to a whole number ≥ 30. To preserve sample size, we kept patients who had a BMI ≥27 with diabetes or 2 or more co-morbidities where weight loss counseling was indicated as per National Institute of Health guidelines[36].

### Measures

#### Body Mass Index

Nursing staff were trained to take height and weight measurements and to calculate BMI. Weight was measured in pounds (rounded to the nearest half pound) using one of 4 calibrated analog scales each equipped with a stadiometer used to take height measurements in inches (rounded to the nearest half inch) with the patients fully clothed except for shoes and jackets. BMI was calculated using the formula BMI = (weight in lbs *703)/(height in inches)^2^.

#### Structured Interview Instrument

We developed a structured interview, conducted by a research assistant and fielded in both English and Spanish, to ensure full and accurate participation of low literacy patients and to assess the core variables of interest [Additional File 1]. We piloted the survey with 20 patients in both Spanish and English to ensure that the questions were understandable, culturally appropriate, and accurately translated into Spanish. Each interview took 30 minutes to complete.

Physicians' use of the 5As of obesity counseling was measured by asking patients whether the physician performed 18 literature-derived 5A's-related counseling skills (e.g. Assess: "Did your doctor ask whether you are currently trying to lose weight?"; Advise: "Did your doctor advise you to lose weight?"; Assist: "did your doctor help you set goals to improve your diet and/or exercise more?") [8,23,25]. The exact questions are published [8, Additional File 1]; the one difference is that we omitted the question "Did you and your doctor discuss your weight today?" since we wanted to focus on quality of counseling. The Cronbach's alpha for these items was 0.89 showing excellent internal consistency. We have also used similiar items in observed structured clinical exams (OSCEs) to evaluate physician performance with adequate internal consistency (Cronbach's alpha = .78).

Finally, we measured theory-based patient, visit, and physician factors that we hypothesized would influence motivation, and intention independent of the quality of physician counseling or that might influence perceptions of the quality of counseling. Patient variables included BMI, gender, primary language, perceived health status, number of co-morbidities, medications, health literacy, self efficacy, and current weight loss, diet and exercise behaviors. Health literacy was assessed using 2 screening questions about ability to read and complete health-related documents [37]. We assessed patients' stage of change by asking participants how true the statement "In the past month, I have been actively trying to lose weight" (or "not gain weight") was of them. We assessed self efficacy to eat healthier and exercise by asking patients to rate their confidence do these things on a 4-pt Likert scale. Current dietary behavior was assessed by averaging responses on 2 statements; "I usually control portions" and "I usually pay attention to fat in my diet" (both using the same 4-point scale). Type of visit was categorized as whether or not this was the patient's first visit with this physician and the interview also assessed whether the physician had advised this patient to lose weight in the past. We measured the patient-centeredness of the physician using six questions adapted from the RAND Corporation Patient Satisfaction Questionnaire [38] and the OPTION (observing patient involvement) shared decision-making scales [39].

Motivation to lose weight was assessed with a direct question --"How motivated are you to lose weight?" [40] and participants responded using a 4-point Likert scale (1 = not at all, 2 = only a little, 3 = somewhat motivated, 4 = very motivated). Intentions were assessed separately for eating and exercise behavior change by asking patients to indicate how true (1 = not at all true, 4 = very true) each of the following statements were of them: "in the next month, I have specific plans to eat healthier" and "in the next month, I have specific plans to exercise". These intention items were developed based on Ajzen's guidelines for writing behavioral intention items in terms of being specific as to target behavior and time frame and then piloted as recommended [41].

For the logistic regression analyses and given that motivations and intentions were both positively skewed, motivation was dichotimized into "very" movivated or less than very motivated and the intention variables were dichotomized into "very true of me" or less (not at all, only a little, somewhat).

### Statistical Analysis

We used means and frequencies to describe our patient sample. Distributions were reported for our dependent variables: motivation and intentions. A "5As counseling score" was calculated as the number of all eighteen 5As skills reported by the patient to have been performed during the visit. We averaged the score of the 7 patient-centeredness items to arrive at a patient-centeredness score. Oneway ANOVAs with Bonferroni post-hoc analysis of pairwise differences were used to assess mean differences in the number of 5As counseling skills by the rated level of motivation and intentions. Finally, logistic regressions were used to explore associations between quality of obesity counseling and the 3 dichotomized outcome variables: whether patients were highly motivated to lose weight, whether it was "very true" that patients had a specific plan to eat better and to exercise regularly in the next month. Potential confounders were included in the logistic regression model and were selected based on whether they were likely to be independently associated with patients' motivation and intentions.

This study was approved by the Institutional Review Boards at New York University School of Medicine and Gouverneur Healthcare Services.

## Results

The nursing staff identified 190 patients through BMI screening. After 28 declined (mostly due to time constraints) and 4 were excluded (non-English or Spanish speaking patients), we interviewed 158 patients of 23 primary care medicine residents from all 3 post-graduate (PGY) years (8 PGY 1, 8 PGY 2, 7 PGY 3). We excluded 4 patients who did not ultimately meet the BMI criteria. Seventeen patients were excluded after the chart abstraction indicated that they had an acute visit, leaving a final sample of 137patients. The mean number of patients per resident was 6.0 (SD = 4.1, range = 1-14).

Table 1 shows the patient characteristics. Of note, the majority of the patients were female and Hispanic. More than half were of low health literacy. Mean self perceived health status was in the fair to good range. More than a third reported actively trying to lose weight and most had high self-efficacy in the areas of eating well and especially exercising regularly. Over half of the visits were first visits and in 29.2% of the visits, the patient had previously been advised to lose weight by this visit's physician.

**Table 1:** Patient, Visit, and Physician Characteristics

	N = 137 obese patients 23 residents
***Sociodemographic Characteristics of Patients***	

% Female (n)	70.1 (96)

Mean Age (SD)	44.9 (13.6)

Race/Ethnicity	

% Latina (n)	77.4(106)

% Black (n)	14.6 (20)

% Low Health Literacy (n)	54.0(74)

% Spanish Speaking (n)	70.1(96)

***Health Characteristics of Patients***	

Mean BMI (SD)	34.2 (4.2)

Mean Self-Report Health Status^1 ^(SD)	3.5 (1.1)

***Stage of Change***	

% Actively Trying to Lose Weight^2 ^(n)	36.5 (50)

***Self-Efficacy***	

% Confident Can Eat Well^3 ^(n)	56.9 (78)

% Confident Can Exercise Regularly^4 ^(n)	80.3 (110)

***Visit Type***	

% New Visit (n)	51.8 (71)

**% Seen this physician once (n)**	10.2 (14)

**% Seen this physician 2-3 times (n)**	23.3 (32)

**% Seen this physician > 3 times (n)**	14.6 (20)

***Physician Characteristics***	

Physician Advised to Lose Weight in Past (%)	29.2(40)

Patient Centeredness^5^	3.8 (0.3)

^1 ^Overall, how would you rate your health during the past four weeks- 6 point scale: Very poor, poor, fair, good, very good, excellent?

^2 ^In the past month, I have been actively trying to lose weight and/or trying not to gain weight.

^3 ^Somewhat or very confident that respondent could choose healthy food, could control the amount of food he/she eats

^4 ^Somewhat or very confident that could exercise regularly

^5 ^Average of six patient centeredness of care items: given every chance to ask questions; physician took a genuine interest in you; gave you information you wanted; checked to make sure you understood everything; gave you opportunity to ask questions; discussed next steps - 4 point scale specific to each item

Table 2 shows patients' responses about their motivation to lose weight and intentions to eat better and get more exercise. The majority of patients were somewhat or very motivated to lose weight and endorsed that they had specific plans to eat healthier and exercise.

**Table 2:** Patient Motivation and Intention to Lose Weight (n = 137)

	Not at All % (n)	Only a Little % (n)	Somewhat % (n)	Very % (n)
**Motivation**				
How motivated are you to make changes related to your weight?	**7.3 (10)**	**10.9 (15)**	**24.8 (34)**	**56.9 (78)**
				
**Intentions**				
How true of you is it that in the next month, you have a specific plan to get more exercise?	**8.0 (11)**	**13.1 (18)**	**29.9 (41)**	**48.9 (67)**
How true of you is it that in the next month, you have a specific plan to eat healthier?	**13.9 (19)**	**10.9 (15)**	**30.6 (42)**	**44.5 (61)**

Table 3 shows the percentage of patients counseled about weight loss, diet, or exercise and the mean number of counseling practices delivered by the physicians (in aggregate and broken down by the 5As). The vast majority of patients received some form of counseling, but the average number of 5As practices was low. "Assess" skills occurred most, followed by Advise practices. Agree, Assist, and Arrange practices were reported to be used, on average, less than once per visit.

**Table 3:** Patient Report of Physician Counseling and Patient Self-Efficacy, Motivation and Intention

	N = 137 patients (23 residents)
**Physician's Use of Obesity Counseling**	

% Counseled about Obesity (n)	85.4 (117)

Mean Number of Counseling Practices^1 ^(SD) (18 Items total)	5.3 (4.6)

**5As**	

Mean Number of **Assess**^2 ^Practices (SD) (6 items)	2.2 (2.0)

Mean Number of **Advise**^3 ^Practices (SD) (4 items)	1.3 (1.3)

Mean Number of **Agree**^4 ^Practices (SD) (3 items)	0.7 (1.2)

% Use **Assist**^5 ^Practice (n) (1 item)	25.6 (35)

Mean Number of **Arrange**^6 ^Practices (SD) (4 items)	0.8 (0.4)

^1 ^Of 18 possible obesity counseling practices (based on 5 As) performed by physician

^2 ^Assessed whether trying to lose weight, importance of losing weight, confidence in losing weight, diet, 24-hour recall of diet, exercise

^3 ^Advised to lose weight, advised how much weight to lose, discussed specific changes in diet and exercise

^4 ^Agreed by working to set goals, involving patient in setting goals, helping to set realistic goals

^5 ^Assisted by talking with patient about dealing with the kinds of things, like stress, temptation, finding time, that makes it hard for you to lose weight

^6 ^Arranged by scheduling follow-up within one month, referring to weight management clinic, community supports like Overeaters Anonymous, or a Nutritionist

Table 4 shows the number of 5As obesity counseling practices by patients' motivation level and intention to eat better and exercise. Based on posthoc follow-up on a significant overall F value with Bonferroni corrections, significant differences in obesity counseling scores were found between those who reported being "only a little" motivated to lose weight and those who were "very motivated" to lose weight. Patients for whom it was "very true" that they had a specific plan to eat better reported significantly more counseling practices than those for whom this statement was less true. In terms of having a plan to exercise regularly, those who felt that this was "somewhat" or "very" true of them reported almost three times as many counseling practices as those who reported that this "not at all" true of them.

**Table 4:** Number of Obesity Counseling Practices by Patients' Motivation Level and Intention to Eat Better and Exercise

Outcomes		Mean Number Counseling Practices (SD)	**F (*p*)**	Post Hoc Difference (Bonferroni)
**Motivation**				
How motivated are you to make changes related to your weight?	Not at All (1)	3.30 (4.11)	F = 3.55 (p = .016)	2<4
	Only a Little (2)	2.50 (2.59)		
	Somewhat (3)	6.12 (4.79)		
	Very (4)	5.47 (4.55)		

**Intentions**				
How true of you is it that in the next month, you have a specific plan to eat better	Not at All (1)	1.36 (1.50)	F = 8.425 (p < .001)	1,2,3<4
	Only a Little (2)	3.11 (3.61)		
	Somewhat (3)	4.61 (4.59)		
	Very (4)	6.93 (4.59)		

How true of you is it that in the next month, you have a specific plan to get more exercise	Not at All (1)	2.26 (3.54)	F = 5.08 (p = .002)	1<3,4
	Only a Little (2)	3.67 (3.81)		
	Somewhat (3)	5.76 (4.43)		
	Very (4)	6.36 (4.69)		

Table 5 shows the results of 3 logistic regression models with dichotomized motivation to lose weight, intention to eat healthier, and intention to exercise each as the dependent variable. Each additional counseling practice was associated with higher odds of being very motivated to lose weight and intending to eat healthier and exercise. Patient centeredness was associated with higher odds of intending to eat better and exercise regularly indicating that how counseling skills are practiced may also be important. Patient and visit characteristics were not significantly associated with motivation and intention, with the exceptions of age (for motivation) and actively trying to lose or maintain weight (for intention to eat better). The odds ratio for self-efficacy to exercise, while not significant, suggests that increases in self-efficacy may be associated with greater intention to exercise.

**Table 5:** Logistic Regressions with Motivation and Intentions as Outcomes (n = 137)

	Motivation			Intention to Eat Better			Intention to Exercise Regularly		
	
Variables	Exp(B)	P	95% CI	Exp(B)	P	95% CI	Exp(B)	P	95% CI
**Patient Characteristics**									

BMI	1.023	.724	.900 - 1.164	1.090	.233	.946 - 1.255	1.087	.227	.949 - 1.246

Self-Report Health Status	.672	.092	.424 - 1.067	1.230	.426	.738 - 2.050	1.469	.155	.864 - 2.495

Female gender	2.158	.177	.707 - 6.583	.971	.961	.303 - 3.107	1.230	.736	.368 - 4.110

Age	.962	.040	.928 - .998	.975	.176	.941 - 1.011	1.000	1.000	.962 - 1.040

Actively Trying to Lose/Not Gain Weight	2.321	.150	.737 - 7.310	4.199	.024	1.208 - 14.601	2.015	.200	.690 - 5.883

Self-Efficacy to Engage in Intended Behavior	n/a	n/a	n/a	1.819	.454	.380 - .874	1.694	.064	.970 - 2.958

**Visit Characteristics**									

New Visit	1.260	.692	.401 - 3.958	.722	.591	.221 - 2.364	.972	.963	.297 - 3.180

Physician Advised Patient to Lose Weight in Past	.770	.699	.204 - 2.902	1.578	.533	.377 - 6.611	2.019	.344	.471 - 8.653

**Physician Characteristics**									

Patient Centeredness	2.029	.374	.426 - 9.655	2.958	.043	1.033 - 8.468	26.076	.001	3.697 - 83.926

Number of Counseling Practices	1.308	.002	1.107 -1.554	1.233	.008	1.056 1.441	1.143	.049	1.001 - 1.305

## Discussion

In this study, we found that resident physicians counseled most of their obese patients about weight. However, they used only a handful of possible 5As skills as part of obesity counseling. Much of the focus of the counseling appeared to involve assessing rather than assisting or arranging. The number of 5As counseling practices reported by the patient to have occurred during the visit was associated with patient motivation to lose weight and intention to change diet and exercise behavior, supporting our hypothesis that higher quality physician counseling may lead to increased motivation and intention to lose weight. An alternative explanation is that patients with higher levels of motivation and intentions are more likely to elicit physician counseling practices and/or report that their physician counseled them. The cross-sectional nature of our study limits our ability to determine the direction of this relationship and future studies are necessary.

If higher quality physician counseling indeed influenced patients' motivation and intentions, then this study highlights theory-based mechanisms through which specific counseling skills can potentially influence patient behavior change. While we were not able to measure actual behavior change or weight loss in this cross-sectional study, we established links between physician counseling practices and important intermediary markers of behavior change. A randomized controlled trial of an intervention to form intentions (or specific plans) in patients attending a commercial weight loss program produced twice the amount of weight loss at 2 months compared with controls [42]. Motivational interviewing, which is done to increase patients' intrinsic motivation to perform a behavior, has been shown to help patients exercise and lose weight [43,44]. By examining motivation and intention, we can better understand potential mechanisms through which physician counseling may play a role in affecting patient behavior.

That the patient-centeredness of the physician was strongly associated with patient intentions suggests that how counseling skills are delivered matters and that quality of the physician/patient relationship may influence the patients' commitment to behavior change. The large odds ratios we found for patient-centeredness may, in part, reflect the lack of variation in this variable which was skewed quite positively and had a low standard deviation. However, we also believe that they reflect the importance of providing patient-centered care in combination with 5As skills to counsel patients to lose weight and change their behaviors. While use of the 5As is considered to be a patient-centered approach to counseling [24], we believe that these are distinct measures since use of the 5As counseling practices leads to higher odds of being motivated and having intentions to change behavior even after controlling for patient centeredness.

There are several limitations to our study. First, generalizability of our findings is limited because we studied a relatively small sample of patients who are mostly underserved, Hispanic patients of physician trainees from a single public clinic. The findings may differ in other populations. Our measure of physicians' use of the 5As is patient-reported and has not been validated with direct observation. The patients' responses may therefore not directly reflect actual physician behavior. Despite this, we believe that patient report and recall of physician counseling may be more important than whether such counseling was directly observed. Also, delivery of the 5As may occur over several visits and it may not be fair to assess quality of physicians' obesity counseling after a single visit. Even more importantly, quality of physician counseling had small effects, in line with those found in similar studies [33] , suggesting that one visit is insufficient for making dramatic differences in obese patients' orientation to behavior change. Finally, this cross sectional study cannot determine causality or assess the directionality of the associations. An alternative interpretation of our results may be that more motivated patients are more likely to receive higher quality obesity counseling. Thus, longitudinal studies and randomized controlled trials are needed to determine whether there is a sustained impact of physician counseling on behavior change in obese patients.

Despite these limitations, this study may inform how we teach physicians to counsel obese patients. Medical school and residency programs already have incorporated patient-centeredness into their curriculums [45-47], and this study supports this approach for obesity counseling. Some authors (including us) propose teaching the 5As to residents for obesity, diet, and exercise counseling [16,24], and we found that each additional 5As-related counseling practice was associated with better outcomes. Future studies should examine the impact of training residents on patient outcomes.

## Conclusions

Use of the 5As counseling practices and delivery of patient-centered care are both markers of quality of obesity counseling. Physicians' use of the 5As  is associated with higher odds of patient motivation to lose weight, intention to eat healthier, and intention to exercise. Patient centeredness is also strongly associated with intentions to change behavior. Future studies need to determine whether higher quality obesity counseling leads to actual changes in patient behavior and weight loss and whether teaching physicians patient-centered care and 5As leads to improvement in patient outcomes.

## Competing interests

The authors declare that they have no competing interests.

## Authors' contributions

All of the authors contributed substantially to this project. MJ designed, implemented, and oversaw all aspects of the study and contributed to the writing of the manuscript. CG participated in the design of the survey, the protocol, analyzed the data, and contributed to writing the manuscript. SS participated in the design and implementation of the study and contributed to writing the manuscript. SS participated in the design of the study and helped to edit the manuscript. AK participated in the design and implementation of the study. She also provided feedback and editing to the manuscript. All the authors have read and approved the final manuscript.

## Pre-publication history

The pre-publication history for this paper can be accessed here:

http://www.biomedcentral.com/1472-6963/10/159/prepub

## Supplementary Material

Additional file 1 **5As Patient Exit Interview Survey**. This survey was read to the patients during the exit interviews. Click here for file
