# Supplementary material for: Physicians' use of the 5As in counseling obese patients: is the quality of counseling associated with patients' motivation and intention to lose weight?
Source: BMC Health Serv Res. 2010 Jun 9;10:159. doi: 10.1186/1472-6963-10-159 (PMC2903583; doi:10.1186/1472-6963-10-159)
Supplement: Additional file 1 — 5As Patient Exit Interview Survey. This survey was read to the patients during the exit interviews. [file 1472-6963-10-159-S1.PDF]

Institutional Review Board
NYU School of Medicine

Veteran's Administration Hospital
Physical Address: 423 East 23rd Street | 10th Floor, West Wing | NY, NY 10010
Mailing Address: 550 1st Avenue | #VET 10th Floor, West Wing | NY, NY 10016

NYU Medical Center

Amendment: Approved

29-Apr-2008

Measuring Patient Outcomes after an Obesity Curriculum for Residents: A Comparison of 3 Different Methods.

Principal Investigator	Dr. Jay, Melanie	Review Type	Expedited [] | Amendment
		Submitted	23-Apr-2008
Phone		Email	melanie.jay@med.nyu.edu
IRB#	08-151	Perf. Period	-
Location(s) Used	OTHER (specified in Overview)		
Review Date	29-Apr-2008	Board/Meeting	Board B on 20-May-2008
Sponsor	HSRA Academic Medicine Fellowship-	Subjects	250 (total) 250 (approved)

The New York University School of Medicine's Institutional Review Board (IRB) is in receipt of your latest submission for the above-referenced study. The submission was reviewed by the IRB on 29-Apr-2008 and the current IRB Status is: Approved.

The following documents are approved for use:

Revised English Patient Survey

Revised Spanish Patient Survey

RE: H#08-151 29-Apr-2008
Elan Czeisler
Director, Institutional Review Board (IRB)
OHRP #FWA00004952

Notes:

1. **You must submit all changes to this study (e.g., *protocol, recruitment materials, consent forms*, etc.) in writing to the IRB for review and approval prior to initiation of the change(s)**, except where necessary to eliminate apparent immediate hazards to the subject(s). Changes made to eliminate apparent immediate hazards to subjects must be reported to the IRB within 24 hours.
2. You must report all adverse and/or unanticipated event(s) that occur during the course of this study to IRB in writing in accordance with IRB Policy.
3. Use only IRB-approved copies of your consent form(s), questionnaire(s), letter(s), advertisement(s), etc. in your study. **Do not use expired consent forms.**
4. You must inform all research staff listed on this study of changes or adverse events which occur.
5. IRB's approval is valid until the end date of the performance period indicated above. A reminder for renewal should be e-mailed to you from the IRB 90, 60 and 30 days before this study's approval is scheduled to expire. However, you are responsible for submitting all renewal materials **at least eight weeks before expiration** regardless of whether or not you receive a reminder notice.
6. All IRB policy documents can be found on our website: http://www.med.nyu.edu/irb/
7. Prior to initiating an IRB-approved study, you must receive written approval from an authorized representative for each site where your study will take place. Key contacts are:
   - **NYU Hospital Center** (Tisch Hospital/Rusk Institute/Co-op Care/HJD/Cancer Center) Kevin Kirchen, VP, Tisch Hospital Administration. 212-263-5806
   - **Bellevue Hospital** Mr. Anand Veereraj, Research Administrator, Bellevue Hospital Research Committee. 212-562-4176; Ms. Setira Simmons, Research Administrator, Bellevue Hospital Research Committee. 212-562-7075
   - **GCRC** (General Clinical Research Center) Hal Rosenblatt, Research Grants Coordinator. 212-263-7900; 212-263-8040

Please Note: Prior to commencement of any research related activities at BHC as a site you MUST receive BHC and HHC approval. For any questions related to research at BHC contact their research office at 212-562-6711 (see above).

The IRB may terminate studies that are not in compliance with NYU Medical Center/School of Medicine Policies & Procedures and the requirements of the Institution's Federal Wide Assurance with the Federal Government. Direct IRB questions, correspondence and forms (e.g., continuing reviews, amendments, adverse events, etc.) to phone 212-263-4110, fax 212-263-4147 or email IRB-info@med.nyu.edu.

New York University School of Medicine's Institutional Review Board
550 First Avenue | #VET 10th Floor, West Wing | New York, NY 10016
phone 212-263-4110 | fax 212-263-4147 | email IRB-info@med.nyu.edu | web http://www.med.nyu.edu/irb
The NYU SoM IRB operates in accordance with Good Clinical Practices (GCP) and applicable laws and regulations

Patient ID #: *Date of Survey:* *Resident ID#:*

draft 7 – 4/11/2008

OBESITY COUNSELING EXIT INTERVIEW

We'd like to ask you some questions about your health care focusing mostly on the visit you just had with your doctor. Everything you tell us will be kept confidential. We're asking patients about their health care so we can help teach doctors to be as effective as possible. The first part of this questionnaire should only take a few minutes....

1. Before we begin, tell me a little bit about the doctor you saw today:

What was his/her name? ☐

Do you consider Dr. ____________________ to be your primary physician? Yes ☐ No ☐

How many times have you seen Dr. ____________________ before today?

None (First Time)	Only Once Before	2 – 3 Times	4 – 10 Times	11 – 20 Times	More than 20 Times
☐	☐	☐	☐	☐	☐

Would you recommend this doctor to a friend or family member?

No	Recommend but With Reservations	Recommend	Highly Recommend
☐	☐	☐	☐

2. For how long have you been getting medical care at Gouverneur?

First Visit	Less than a month	1 – 6 mos	6+ months	a year or more	up to 5 yrs	5+ yrs
☐	☐	☐	☐	☐	☐	☐

GENERAL SATISFACTION WITH HEALTH CARE AT GOUVERNEUR

The next few questions focus on the medical care you've generally received at Gouverneur.

(If this is your first visit at Gouverneur, answer these questions thinking about this visit.)

3. How satisfied are you with the medical care you receive at Gouverneur?

Not at all Satisfied	Only a Little Satisfied	Somewhat Satisfied	Very Satisfied
☐	☐	☐	☐

4. How often are the people who treat you at Gouverneur in too much of a hurry?

Never	Once in A While	Sometimes	Always
☐	☐	☐	☐

5. How hard is it to get an appointment for medical care when you need it?

Not at all Hard (it's easy)	Only a Little Bit Hard	Somewhat Hard	Very Hard (it's impossible)
☐	☐	☐	☐

6. How long do you usually have to wait to see the doctor once you get to Gouverneur?

Not Long At All	Only a Little Bit Long	Somewhat Long	Much Too Long
☐	☐	☐	☐

7. Do staff members at Gouverneur treat you with courtesy and respect?

No (they're disrespectful)	Only a Little Respectful	Somewhat Respectful	Very Respectful
☐	☐	☐	☐

8. How careful are the doctors to check everything when they treat and examine you?

Not at All Careful	Only a Little Careful	Somewhat Careful	Very Careful
☐	☐	☐	☐

NYUSOM
IRB APPROVED
04/29/2008

INFORMATION ABOUT ***THIS VISIT***
I'd like to ask you a few questions about the visit you just finished....

9. What did you and your doctor talk about today? *[PROBE: Anything else?]*

10. How satisfied are you with your visit with your doctor today?

Not at all Satisfied	Only a Little Satisfied	Somewhat Satisfied	Very Satisfied
☐	☐	☐	☐

11. Did the doctor give you every chance to talk about your problems or concerns?

No (didn't let me talk)	Only a Little (only gave a little bit of a chance to talk)	Somewhat of A Chance	Very Much of a Chance (gave me every chance)
☐	☐	☐	☐

12. Did the doctor show a genuine interest in you?

No (no interest in me)	Only A Little Interest	Some Interest in Me	Very Much Interested in Me
☐	☐	☐	☐

13. Did the doctor give you the information you wanted?

Not at All	Only Gave Me a Little of the Info I Wanted	Some of the Info I Wanted	Gave Me All of the Info I Wanted
☐	☐	☐	☐

14. Did the doctor check to make sure you understood everything?

No, didn't check at all	Checked at least 1 thing	Checked for some things	Checked everything
☐	☐	☐	☐

15. Did the doctor give you opportunities to ask all of the questions you had?

No (I wasn't able to ask ?s)	I was only able to ask a few questions	I was able to ask some of my questions	I was able to ask all of my questions
☐	☐	☐	☐

16. Did the doctor ask you what thought about the next steps regarding your care?

No, didn't ask me at all	Only asked me if I had ?s	Asked me what I thought about some of next steps	Asked me what I thought about all of the next steps
☐	☐	☐	☐

INFORMED CONSENT PROCESS FOR PATIENT

INFORMATION ABOUT ***YOU***
Now that we've talked a lot about your doctor, we'd like to ask some questions about you so that we can better understand how doctors work with different patients. These questions will focus a lot on diet and exercise and weight management because that's one of the areas where we want to try and improve doctors' effectiveness.

17. Tell us a little bit about your plans. How true is each of the following statements...?

	Not at All True of Me	Only a Little True	Somewhat True	Very True of Me
In the next month, I have a specific plan to get more exercise	☐	☐	☐	☐
In the next month, I have a specific plan to eat healthier	☐	☐	☐	☐

18. How true is each of the following statements of you…

	Not at All True of Me	Only a Little True	Somewhat True	Very True of Me
In the past month, I have been actively trying to lose weight	☐	☐	☐	☐
In the past month, I have been actively trying to keep from gaining weight	☐	☐	☐	☐
Are you seriously thinking about trying to reach your target weight in the next 6 months?	☐	☐	☐	☐
Have you maintained your desired weight for more than 6 months?	☐	☐	☐	☐

19. Did you and your doctor discuss your weight today?

☐ No ☐ Yes → What did you discuss?

→How much of the visit was spent talking about weight?

Only a Little Bit of Visit	Some of the Visit	Most of the Visit	All of the Visit
☐	☐	☐	☐

20. Did your doctor tell you that you should lose weight?

☐ No ☐ Yes → Did you discuss how much weight you should lose?

→ ☐ No ☐ Yes → How much weight? ________lbs

Do you think you can lose that much weight? ☐ No ☐ Yes

21. Has this doctor ever advised you to lose weight in the past?

☐ No ☐ Yes →Did he/she follow up on your past conversation regarding your weight? ☐ No ☐ Yes

→ Has any doctor advised you to lose weight in the past? ☐ No ☐ Yes

22. Did your doctor ask you any of the following?

		No	Yes	***I Don't Remember***
a	if you're trying to lose weight?	☐	☐	☐
b	how important you think it is to try and lose weight?	☐	☐	☐
c	you how confident you are that you can lose weight?	☐	☐	☐

23. Did your doctor ask you about or discuss your diet (what you eat) today?

☐ No ☐ Yes → Did your doctor discuss making changes in your diet with you today?

→☐ No ☐ Yes→ What changes?

23. b. Did you doctor ask you to list or write down everything you have eaten in the past twenty-four hours or in a typical day?

☐ No ☐ Yes

24. Did your doctor ask you about or discuss exercise today?

☐ No ☐ Yes → Did your doctor discuss making changes in how much you exercise?

→ ☐ No ☐ Yes→ What changes?

25. Did your doctor help you set goals (make specific plans) to improve your diet, exercise more?

☐ No ☐ Yes → What specific plans or goals did you make/set?

How much were you involved in setting these goals?

Not at All	Only a Little	Somewhat	Very Involved
☐	☐	☐	☐

Do you think it's realistic that you'll meet these goals?

Not at All	Only a Little	Somewhat	Very Involved
☐	☐	☐	☐

26. Did your doctor talk with you about how to deal with the kinds of things, like stress, temptation, finding time, that make it hard for you to lose weight (exercise, eat healthy)?

Not at All	Only a Little	Somewhat	A lot
☐	☐	☐	☐

What kinds of things did you discuss?

27. How motivated are you to make changes related to your weight?

Not at All	Only a Little	Somewhat	Very Motivated
☐	☐	☐	☐

28. Did your doctor tell you when he or she wanted to see you again to follow up?

☐ No ☐ Yes →When did he/she tell you to come back? ☐ within a month ☐ 1 – 3 months ☐ 4 – 6 months ☐ 1 year

29. Did your doctor refer you to any of the following?

	No	Yes	I Don't Remember
weight management clinic	☐	☐	☐
community supports like Overeaters Anonymous	☐	☐	☐
nutritionist	☐	☐	☐
other *SPECIFY* ______________________	☐	☐	☐

30. Please indicate how much you agree or disagree with how true each of the following statements is for you:

		Strongly Disagree	Somewhat Disagree	Somewhat Agree	Strongly Agree	Not Appli cable
a	When all is said and done, I am the person who is responsible for managing my weight problem	☐	☐	☐	☐	☐
b	Taking an active role in my own health care is the most important factor in determining my health and ability to function	☐	☐	☐	☐	☐
c	I am confident that I can take actions that will help prevent or minimize some symptoms or problems associated with my weight	☐	☐	☐	☐	☐
d	I am confident that I can tell when I need to go get medical care and when I can handle a health problem myself	☐	☐	☐	☐	☐
e	I am confident I can tell my health care provider concerns I have even when he or she does not ask	☐	☐	☐	☐	☐
f	I am confident that I can follow through on the diet and exercise recommendations my doctor makes	☐	☐	☐	☐	☐
g	I understand the nature and causes of my weight problem	☐	☐	☐	☐	☐
h	I know the different treatment options available for my weight problem	☐	☐	☐	☐	☐
i	I have been able to maintain the lifestyle changes that I have made in the past	☐	☐	☐	☐	☐
j	I am confident that I can maintain lifestyle changes, like diet and exercise, even during times of stress	☐	☐	☐	☐	☐
k	I am confident that I can figure out solutions when new situations or problems arise with my weight	☐	☐	☐	☐	☐
l	I exercise regularly	☐	☐	☐	☐	☐
m	I usually pay attention to fat in my diet	☐	☐	☐	☐	☐
n	I usually control portions (eat less of what's on my plate)	☐	☐	☐	☐	☐
o	I eat plenty of fruits and vegetables every day	☐	☐	☐	☐	☐

31. Please tell me how confident you are you that you could do the following:

		Not at All Confident	Only a Little Confident	Somewhat Confident	Very Confident	Not Appli cable
a	I could chose healthy food that would help me lose weight	☐	☐	☐	☐	☐
b	I could exercise (e.g., walking) regularly (most days of the week)	☐	☐	☐	☐	☐
c	I could control the amount of food I eat	☐	☐	☐	☐	☐

32. Overall, how would you rate your health during the past 4 weeks?

Very Poor	Poor	Fair	Good	Very Good	Excellent
☐	☐	☐	☐	☐	☐

33. During the past 4 weeks, how much....

		Not at All	Very Little	Somewhat	Quite a lot	Not Appli cable
a	did your physical health problems limit your usual physical activities (such as walking or climbing stairs)?	☐	☐	☐	☐	☐
b	difficulty did you have doing your daily work, both at home and away from home, because of your physical health?	☐	☐	☐	☐	☐

34. How much bodily pain have you had during the past four weeks?

None	Very Mild	Mild	Moderate	Severe	Very Severe
☐	☐	☐	☐	☐	☐

35. During the past four weeks, how much energy did you have?

None	A Little	Some	Quite a Lot	Very Much
☐	☐	☐	☐	☐

36. During the past four weeks, how much did your physical health or emotional problems limit your usual social activities with family or friends?

Not at All	Very Little	Somewhat	Quite a Lot	Very Much
☐	☐	☐	☐	☐

37. During the past four weeks, how much have you been bothered by emotional problems (such as feeling anxious, depressed or irritable)?

Not at All	Slightly	Moderately	Quite a Lot	Extremely
☐	☐	☐	☐	☐

38. During the past four weeks, how much of the time have you felt downhearted or depressed?

None of the Time	A Little	Some	Most of the Time	All of the Time
☐	☐	☐	☐	☐

39. During the past four weeks, how much did personal or emotional problems keep you from doing your usual work, school, or other daily activities?

Not at All	Slightly	Moderately	Quite a Lot	Extremely
☐	☐	☐	☐	☐

40. Do you have any of the following conditions?

obesity	☐ No	☐ Not Sure	☐ Yes	→ Do you take any medications for this?	☐ No	☐ Yes
diabetes	☐ No	☐ Not Sure	☐ Yes	→ Do you take any medications for this?	☐ No	☐ Yes
high cholesterol	☐ No	☐ Not Sure	☐ Yes	→ Do you take any medications for this?	☐ No	☐ Yes
high blood pressure	☐ No	☐ Not Sure	☐ Yes	→ Do you take any medications for this?	☐ No	☐ Yes
arthritis	☐ No	☐ Not Sure	☐ Yes	→ Do you take any medications for this?	☐ No	☐ Yes

a. How often do you take your medications as indicated?

Not at All	A Little of Time	Some of the Time	All of the Time
☐	☐	☐	☐

b. How well are you doing in managing this (these) conditions?

Not at all Well	Only A Little Bit Well	Somewhat Managing	Managing Very Well
☐	☐	☐	☐

Do you take any other medications?	☐ No	☐ Yes	→What medications? What for?

41. How often do you have someone help you read materials or pamphlets or information related to your health or health care in English?

Never	Occasionally	Sometimes	Often	Always
☐	☐	☐	☐	☐

42. How confident are you filling out medical and health forms by yourself in English?

Not at All	Only a Little	Somewhat	Quite Confident	Extremely
☐	☐	☐	☐	☐

A FEW LAST QUESTIONS ABOUT YOUR BACKGROUND....

43. Are you ...

- Single ☐
- Married or in marriage-like relationship ☐
- Separated/Divorced ☐
- Widowed ☐
- *OTHER* ☐ *Specify:* ____________________

44. What level of school did you complete?

- less than high school ☐
- high school or GED ☐
- college ☐
- graduate school ☐

45. What language do you consider to be your primary language (e.g., spoken the most in your household)?

- English ☐
- Spanish ☐
- Both English and Spanish ☐

46. What language was used in this visit? [Probe: English? Spanish? Both?]

____

47. What do you think was your doctor's primary language? (If don't know, what's your best guess?)

____

48. Do you identify yourself as Hispanic or Latino/a? ☐ No ☐ Yes

49. How would you classify ...

***your* race/ethnicity? Are you...?**

- Black or African or Caribbean American ☐
- White or Caucasian ☐
- Asian ☐
- American Indian or Alaska Native ☐
- *OTHER:* ________________________ ☐

N.º de identificación del paciente *Fecha de entrevista* *N.º de identificación del residente*

Versión 7 – 2/4/2008

ENTREVISTA FINAL SOBRE EL SERVICIO DE ASESORAMIENTO PARA LA OBESIDAD

Nos gustaría hacerle algunas preguntas sobre su atención médica, centrándonos especialmente en la visita que ha realizado a su médico. Todo lo que nos diga será confidencial. Estamos consultando a los pacientes sobre el servicio de atención médica para lograr que los médicos sean más eficaces. La primera parte de este cuestionario solo durará unos pocos minutos...

1. Antes de comenzar, por favor cuénteme un poco acerca del médico que visitó hoy:

¿Cómo se llamaba? ☐

¿Usted considera que el Dr. /la Dra.__________ es su médico principal? **Sí** ☐ **No** ☐

¿Cuántas veces ha visitado al Dr./a la Dra. ________________ antes de la visita de hoy?

Ninguna (Primera vez)	Solo una vez	2 – 3 veces	4 – 10 veces	11 – 20 veces	Más de 20 veces
☐	☐	☐	☐	☐	☐

¿Le recomendaría este médico a un amigo o miembro de su familia?

No	Lo recomendaría pero con reservas	Lo recomendaría	Sí, es muy recomendable
☐	☐	☐	☐

2. ¿Cuánto hace que está recibiendo servicios médicos en Gouverneur?

Primera visita	Menos de un mes	1 – 6 meses	Más de 6 meses	Un año o más	hasta 5 años	Más de 5 años
☐	☐	☐	☐	☐	☐	☐

GRADO DE SATISFACCIÓN GENERAL CON LA ATENCIÓN MÉDICA RECIBIDA EN GOUVERNEUR
Las próximas preguntas se centran en la atención médica que ha recibido en Gouverneur.
(Si esta es su primera visita en Gouverneur, por favor responda a estas preguntas pensando en esta visita).

3. ¿Qué tan satisfecho(a) está con la atención médica que recibió en Gouverneur?

Para nada satisfecho(a)	Solo un poco satisfecho(a)	Algo satisfecho(a)	Muy satisfecho(a)
☐	☐	☐	☐

4. ¿Con qué frecuencia ha notado que las personas que lo atienden en Gouverneur tienen mucha prisa?

Nunca	De vez en cuando	A veces	Siempre
☐	☐	☐	☐

5. ¿Qué tan difícil es pedir una cita médica para recibir atención médica cuando la necesita?

Para nada difícil (es fácil)	Solo un poco difícil	Algo difícil	Muy difícil (es imposible)
☐	☐	☐	☐

6. ¿Cuánto tiempo debe esperar normalmente para ver al médico una vez que está en Gouverneur?

Nada	Solo un poco	Algo	Mucho
☐	☐	☐	☐

7. ¿Considera que el personal de Gouverneur lo trata con cortesía y respeto?

No (son irrespetuosos)	Son solo un poco respetuosos	Son algo respetuosos	Son muy respetuosos
☐	☐	☐	☐

NYUSOM
IRB APPROVED
04/29/2008

8. ¿Qué tan cuidadosos son los médicos de examinar todos los aspectos cuando lo(a) están tratando y examinando?

Para nada cuidadosos	Solo un poco cuidadosos	Algo cuidadosos	Muy cuidadosos
☐	☐	☐	☐

INFORMACIÓN SOBRE ***ESTA VISITA***
Me gustaría hacerle algunas preguntas sobre la visita que ha realizado hoy...

9. ¿De qué habló con su médico hoy? *[INDAGAR: ¿Algo más?]*

10. ¿Qué tan satisfecho(a) está con su visita médica hoy?

Para nada satisfecho(a)	Solo un poco satisfecho(a)	Algo satisfecho(a)	Muy satisfecho(a)
☐	☐	☐	☐

11. ¿El médico le dio la posibilidad de hablar sobre sus problemas o preocupaciones?

No (no me dejó hablar)	Solo un poco (me dio muy pocas posibilidades)	Me dio algunas posibilidades	Sí (me dio todas las posibilidades)
☐	☐	☐	☐

12. ¿Considera que el médico mostró un interés genuino en usted?

No (no mostró interés en mí)	Solo un poco de interés	Mostró algo de interés	Mostró mucho interés en mí
☐	☐	☐	☐

13. ¿Considera que el médico le dio toda la información que usted esperaba?

Para nada	Solo me dio una parte de la información que esperaba	Me dio algo de la información que esperaba	Me dio toda la información que esperaba
☐	☐	☐	☐

14. ¿El médico verificó que usted hubiera entendido todo?

No, no lo verificó	Verificó al menos 1 punto	Verificó algunos puntos	Verificó todo
☐	☐	☐	☐

15. ¿El médico le dio la posibilidad de hacer todas las preguntas que deseaba?

No (no tuve la posibilidad de preguntar)	Pude hacer muy pocas preguntas	Solo pude hacer algunas de mis preguntas	Pude hacer todas mis preguntas
☐	☐	☐	☐

16. ¿El médico le preguntó qué pensaba sobre sus planes para el futuro con respecto a su atención médica?

No, no me preguntó nada	Solo me preguntó si yo tenía preguntas	Me preguntó qué pensaba sobre algunos de mis planes para el futuro	Me preguntó qué pensaba sobre todos mis planes para el futuro
☐	☐	☐	☐

PROCESO DE CONSENTIMIENTO INFORMADO DEL PACIENTE

INFORMACIÓN SOBRE ***USTED***

Ahora que ya hemos hablado bastante sobre su médico, me gustaría hacerle algunas preguntas personales para que podamos comprender mejor de qué forma los médicos trabajan con los distintos pacientes. Estas preguntas se centrarán principalmente en su dieta y ejercicios y en el control de peso porque esa es una de las áreas en las cuales queremos mejorar la eficacia de los médicos.

17. Cuéntenos un poco sobre sus planes. ¿Qué grado de certeza posee cada una de las siguientes afirmaciones...?

	**No es cierto**	**Un poco cierto**	**Algo cierto**	**Muy cierto**
Para el próximo mes, tengo un plan específico para hacer más ejercicio	☐	☐	☐	☐
Para el próximo mes, tengo un plan específico para alimentarme de forma más saludable	☐	☐	☐	☐

18. ¿Qué grado de certeza posee cada una de las siguientes afirmaciones sobre usted...?

	**No es cierto**	**Un poco cierto**	**Algo cierto**	**Muy cierto**
El último mes, he intentado bajar de peso activamente	☐	☐	☐	☐
El último mes, he intentado no engordar activamente	☐	☐	☐	☐
¿Ha pensado seriamente en intentar alcanzar su meta respecto a su peso en los próximos 6 meses?	☐	☐	☐	☐
¿Ha logrado mantener su peso deseado por más de 6 meses?	☐	☐	☐	☐

19. ¿Habló con su médico acerca de su peso en la visita de hoy?

☐ No ☐ Sí → ¿Sobre qué hablaron?

	**Solo parte de la visita**	**Algo de la visita**	**La mayor parte de la visita**	**Toda la visita**
→¿Cuánto tiempo de la visita se dedicó a hablar sobre el peso?	☐	☐	☐	☐

20. ¿Su médico mencionó que usted debería bajar de peso?

☐ No ☐ Sí → ¿Hablaron sobre cuántas libras debería bajar?

→ ☐ No ☐ Sí → ¿Cuántas libras? ________libras

¿Cree que puede bajar esa cantidad de libras? ☐ No ☐ Sí

21. ¿En el pasado, este médico le aconsejó que bajara de peso?

☐ No ☐ Sí →¿Habló de su conversación previa sobre el peso? ☐ No ☐ Sí

→ ¿Alguna vez un médico le aconsejó que bajara de peso? ☐ No ☐ Sí

22. ¿El médico le hizo alguna de las siguientes preguntas?

		No	Sí	No me acuerdo
a.	¿El médico le preguntó si usted está tratando de bajar de peso?	☐	☐	☐
b.	¿El médico le preguntó qué importancia cree que tiene intentar bajar de peso?	☐	☐	☐
c.	¿El médico le preguntó qué tan seguro(a) se siente de poder bajar de peso?	☐	☐	☐

23. ¿El médico le preguntó o le habló sobre su dieta (lo que usted come) en la visita de hoy?

☐ No ☐ Sí → ¿Su médico habló con usted sobre cambios específicos que debe hacer en su dieta en la visita de hoy?

☐ No ☐ Sí→ ¿Cuáles son los cambios?

23. b. El medicó le preguntó decir o escribir toda lo que ha comido en las ultimas 24 horas o durante un día típica?

☐ No ☐ Yes

24. ¿El médico le preguntó o le habló sobre actividad física en la visita de hoy?

☐ No ☐ Sí → ¿Su médico mencionó cambios específicos en la cantidad de actividad física?

☐ No ☐ Sí→ ¿Cuáles son los cambios?

25. ¿El médico le ayudó a fijar metas específicas para mejorar su dieta y hacer más ejercicio?

☐ No ☐ Sí → ¿Cuáles son?

¿Qué participación tuvo usted en la fijación de estas metas?

Ninguna	Poca	Alguna	Mucha
☐	☐	☐	☐

¿Considera estas metas realistas?

Para nada	Un poco	Algo	Muy
☐	☐	☐	☐

26. ¿El médico mencionó la forma de sobrellevar algunas situaciones (el estrés, la tentación, la falta de tiempo) que hacen que pueda ser difícil para usted bajar de peso (hacer ejercicio, alimentarse saludablemente)?

Para nada	Un poco	Algo	Mucho
☐	☐	☐	☐

~~**¿Por ejemplo?**~~

27. ¿Qué tan motivado(a) se encuentra para realizar las modificaciones relacionadas con su peso?

Nada	Un poco	Algo	Muy motivado
☐	☐	☐	☐

28. ¿El médico mencionó cuándo quería verlo de nuevo para hacer un seguimiento?

☐ No ☐ Sí →¿En cuánto tiempo le pidió que regresara?

☐ dentro de un mes	☐ 1 – 3 meses	☐ 4 – 6 meses	☐ 1 año

29. ¿El médico le recomendó o le dio información sobre alguno de los siguientes servicios?

	No	Sí	No me acuerdo
clínica de control de peso	☐	☐	☐
grupos de apoyo comunitarios, como Comedores Compulsivos Anónimos	☐	☐	☐
nutricionista	☐	☐	☐
otro *DETALLAR*______________________________	☐	☐	☐

30. Por favor, indique su acuerdo o desacuerdo con el grado de certeza de cada una de las siguientes afirmaciones sobre usted:

		Totalmente en desacuerdo	Algo en desacuerdo	Algo de acuerdo	Totalmente de acuerdo	No corres ponde
A	Cuando está todo dicho, yo soy la persona encargada de controlar mis problemas de peso	☐	☐	☐	☐	☐
B	Lograr la participación activa en mi propia atención médica es el factor más importante para tomar decisiones sobre mi salud y mi capacidad de desempeño	☐	☐	☐	☐	☐
C	Estoy seguro(a) de que puedo tomar medidas que me ayudarán a evitar o a minimizar algunos síntomas o problemas asociados con mi peso	☐	☐	☐	☐	☐
D	Estoy seguro(a) de que puedo darme cuenta del momento en que debo obtener atención médica y cuándo puedo manejar un problema de salud solo	☐	☐	☐	☐	☐
E	Estoy seguro(a) de que puedo transmitirle a mi médico las preocupaciones que tengo aunque este no me pregunte nada	☐	☐	☐	☐	☐
F	Estoy seguro(a) de que puedo cumplir con las recomendaciones de dieta y ejercicios realizadas por mi médico	☐	☐	☐	☐	☐
G	Comprendo la naturaleza y las causas de mis problemas de peso	☐	☐	☐	☐	☐
H	Conozco las diferentes opciones de tratamiento disponibles para tratar mis problemas de peso	☐	☐	☐	☐	☐
I	He podido mantener los cambios en mi estilo de vida realizados en el pasado	☐	☐	☐	☐	☐
J	Estoy seguro(a) de que puedo mantener los cambios en mi estilo de vida, por ejemplo cosas asociadas con mi dieta y ejercicio, aún en momentos de estrés	☐	☐	☐	☐	☐
K	Estoy seguro(a) de que puedo encontrar soluciones cuando se presenten nuevos problemas o situaciones relacionados con mi peso	☐	☐	☐	☐	☐
L	Practico ejercicio con regularidad	☐	☐	☐	☐	☐
M	Normalmente presto atención al contenido de grasas de mi dieta	☐	☐	☐	☐	☐

		Totalmente en desacuerdo	Algo en desacuerdo	Algo de acuerdo	Totalmente de acuerdo	No corres ponde
N	Normalmente controlo las porciones (como menos de lo que hay en mi plato)	☐	☐	☐	☐	☐
O	Como muchos vegetales y frutas todos los días	☐	☐	☐	☐	☐

31. Por favor, indique qué tan seguro(a) se siente de poder hacer las siguientes cosas:

		Para nada seguro (a)	Un poco seguro (a)	Algo seguro(a)	Muy seguro(a)	No corres ponde
a.	Podría elegir alimentos saludables que me ayudarían a bajar de peso	☐	☐	☐	☐	☐
b.	Podría hacer ejercicio (caminar) regularmente (casi todos los días de la semana)	☐	☐	☐	☐	☐
c.	Podría controlar la cantidad de alimentos que ingiero	☐	☐	☐	☐	☐

32. En términos generales ¿cómo valoraría su salud durante las últimas 4 semanas?

Muy mala	Mala	Regular	Buena	Muy buena	Excelente
☐	☐	☐	☐	☐	☐

33. Durante las últimas 4 semanas...

		Nada	Un poco	Algo	Bastante	No corres ponde
a.	¿En qué medida sus problemas de salud físicos limitaron sus actividades físicas normales (como caminar o subir las escaleras)?	☐	☐	☐	☐	☐
b.	¿Qué grado de dificultad tuvo al cumplir con sus trabajos diarios, tanto en su hogar como fuera de casa, a causa de su salud física?	☐	☐	☐	☐	☐

34. ¿Qué grado de dolor corporal sintió en las últimas cuatro semanas?

Ninguno	Muy leve	Leve	Moderado	Intenso	Muy intenso
☐	☐	☐	☐	☐	☐

35. Durante las últimas 4 semanas, ¿cuánta energía sintió?

Nada	Un poco	Algo	Bastante	Mucha
☐	☐	☐	☐	☐

36. Durante las últimas 4 semanas, ¿de qué forma su salud física o los problemas emocionales dificultaron sus actividades sociales con amigos o parientes?

Para nada	Muy poco	Algo	Bastante	Mucho
☐	☐	☐	☐	☐

37. Durante las últimas 4 semanas, ¿Qué tan preocupado(a) se ha sentido por problemas emocionales (sentimientos de ansiedad, depresión o irritabilidad)?

Nada	Un poco	Moderadamente	Bastante	Extremadamente
☐	☐	☐	☐	☐

38. Durante las últimas 4 semanas, ¿cuánto tiempo se ha sentido desanimado(a) o deprimido(a)?

Nada	Un poco	Algo	Casi todo el tiempo	Todo el tiempo
☐	☐	☐	☐	☐

39. Durante las últimas 4 semanas, ¿los problemas personales o emocionales le impidieron realizar su trabajo, asistir al colegio u otras actividades diarias normales?

Para nada	Un poco	Moderadamente	Bastante	Extremadamente
☐	☐	☐	☐	☐

40. ¿Tiene alguna de las siguientes condiciones?

obesidad	☐ No	☐ No estoy seguro	☐ Sí	→ ¿Toma algún medicamento?	☐ No	☐ Sí
diabetes	☐ No	☐ No estoy seguro	☐ Sí	→ ¿Toma algún medicamento?	☐ No	☐ Sí
colesterol alto	☐ No	☐ No estoy seguro	☐ Sí	→ ¿Toma algún medicamento?	☐ No	☐ Sí
hipertensión	☐ No	☐ No estoy seguro	☐ Sí	→ ¿Toma algún medicamento?	☐ No	☐ Sí
artritis	☐ No	☐ No estoy seguro	☐ Sí	→ ¿Toma algún medicamento?	☐ No	☐ Sí

a. ¿Con qué frecuencia toma sus medicamentos según lo indicado?

Nunca	En alguna ocasión	Algunas veces	Siempre
☐	☐	☐	☐

b. ¿Qué tan bien le va en el manejo de estas condiciones?

Para nada bien	Un poco bien	Más o menos bien	Muy bien
☐	☐	☐	☐

¿Toma otros medicamentos?	☐ No	☐ Sí	→¿Qué medicamento(s)? ¿Para qué?

41. ¿Con qué frecuencia necesita alguien que le ayuda a leer material, folletos o información relacionada con su salud o con su atención médica cuando esos materiales están en español?

Nunca	Ocasionalmente	A veces	Con frecuencia	Siempre
☐	☐	☐	☐	☐

41.b. ¿Con qué frecuencia necesita alguien que le ayude a leer material, folletos o información relacionada con su salud o con su atención médica cuando esos materiales están en inglés?

Nunca	Ocasionalmente	A veces	Con frecuencia	Siempre
☐	☐	☐	☐	☐

42. ¿Se siente seguro(a) al completar los formularios médicos y de salud sin ayuda cuando están en español?

Para nada	Un poco	Algo	Bastante seguro	Extremadamente
☐	☐	☐	☐	☐

42. b. ¿Se siente seguro(a) al completar los formularios médicos y de salud sin ayuda cuando están en inglés?

Para nada	Un poco	Algo	Bastante seguro	Extremadamente
☐	☐	☐	☐	☐

ÚLTIMAS PREGUNTAS SOBRE USTED...

43. Usted es...

Soltero(a)	☐	
Casado(a) o relación similar	☐	
Separado(a)/Divorciado(a)	☐	
Viudo(a)	☐	
OTRO	☐	*Detallar:* ____________________

44. ¿Qué nivel de educación ha alcanzado?

menos de educación secundaria	☐
educación secundaria o GED	☐
educación universitaria	☐
estudios de posgrado	☐

45. ¿Cuál considera que es su idioma principal (el que se habla con más frecuencia en su hogar)?

____

46. ¿Qué idioma se utilizó durante esta visita? [Indagar: ¿inglés? ¿español? ¿ambos?]______________________

47. ¿Cuál cree que es el idioma principal de su médico? ____________________________________

48. ¿Usted se siente identificado como hispano(a) o latino(a)? ☐ No ☐ Sí

49. ¿Cómo clasificaría...

***Su* raza/etnia? Usted es...**	
De raza negra, afroamericana o caribeño-americana	☐
Blanco o caucásico	☐
Asiático	☐
Indio americano o nativo de Alaska	☐
OTRO(A): ________________________	☐
